# Analysis of Mrgprb2 Receptor-Evoked Ca^*2*+^ Signaling in Bone Marrow Derived (BMMC) and Peritoneal (PMC) Mast Cells of TRPC-Deficient Mice

**DOI:** 10.3389/fimmu.2020.00564

**Published:** 2020-04-08

**Authors:** Volodymyr Tsvilovskyy, Alejandra Solis-Lopez, Julia Almering, Christin Richter, Lutz Birnbaumer, Alexander Dietrich, Marc Freichel

**Affiliations:** ^1^Pharmakologisches Institut, Ruprecht-Karls-Universität Heidelberg, Heidelberg, Germany; ^2^Neurobiology Laboratory, National Institute of Environmental Health Sciences, Durham, NC, United States; ^3^Institute for Biomedical Research (BIOMED), Catholic University of Argentina, Buenos Aires, Argentina; ^4^Walther-Straub Institut für Pharmakologie und Toxikologie, Ludwig-Maximilians-Universität München, Munich, Germany

**Keywords:** mast cells degranulation, secretagogues, connective tissue type mast cells, mucosal tissue type mast cells, TRPC channels, Mrgprb2 receptor, intracellular calcium

## Abstract

Mast cells are a heterogeneous group of immune cells. The simplest and commonly accepted classification divides them in two groups according to their protease content. We have compared the action of diverse secretagogues on bone marrow derived (BMMC) and peritoneal (PMC) mast cells which represent classical models of mucosal and connective tissue type mast cells in mice. Whereas, antigen stimulation of the FcεRI receptors was similarly effective in triggering elevations of free intracellular Ca^2+^ concentration ([Ca^2+^]_i_) in both BMMC and PMC, robust [Ca^2+^]_i_ rise following Endothelin-1 stimulation was observed only in a fraction of BMMC. Leukotriene C4 activating cysteinyl leukotriene type I receptors failed to evoke [Ca^2+^]_i_ rise in either mast cell model. Stimulation of the recently identified target of many small-molecule drugs associated with systemic pseudo-allergic reactions, Mrgprb2, with compound 48/80, a mast cell activator with unknown receptor studied for many years, triggered Ca^2+^ oscillations in BMMC and robust [Ca^2+^]_i_ rise in PMCs similarly to that evoked by FcεRI stimulation. [Ca^2+^]_i_ rise in PMC could also be evoked by other Mrgprb2 agonists such as Tubocurarine, LL-37, and Substance P. The extent of [Ca^2+^]_i_ rise correlated with mast cell degranulation. Expression analysis of TRPC channels as potential candidates mediating agonist evoked Ca^2+^ entry revealed the presence of transcripts of all members of the TRPC subfamily of TRP channels in PMCs. The amplitude and AUC of compound 48/80-evoked [Ca^2+^]_i_ rise was reduced by ~20% in PMC from *Trpc1/4/6*^−/−^ mice compared to *Trpc1/4*^−/−^ littermatched control mice, whereas FcεRI-evoked [Ca^2+^]_i_ rise was unaltered. Whole-cell patch clamp recordings showed that the reduction in compound 48/80-evoked [Ca^2+^]_i_ rise in *Trpc1/4/6*^−/−^ PMC was accompanied by a reduced amplitude of Compound 48/80-induced cation currents which exhibited typical features of TRPC currents. Together, this study demonstrates that PMC are an appropriate mast cell model to study mechanisms of Mrgprb2 receptor-mediated mast cell activation, and it reveals that TRPC channels contribute at least partially to Mrgprb2-mediated mast cellactivation but not following FcεRI stimulation. However, the channels conducting most of the Ca^2+^ entry in mast cells triggered by Mrgprb2 receptor stimulation remains to be identified.

## Introduction

Mast cells play a very important role in innate and adaptive immunity by their capability of quick and massive release of granules containing preformed inflammatory mediators and proteases (1) as well as by the capability to secrete a broad spectrum of cytokines and growth factors (2). The canonical way of mast cell activation is their participation in an immediate allergic reaction of anaphylactic type involving a specific antigen-induced crosslinking of surface IgE molecules bound to high-affinity receptors for IgE (FcεRI). However, in clinical practice there are a lot of examples of mast cell participation in less specific pseudoallergic reactions. Recently, a pivotal role of a Mrgpr in initiation of such reactions was shown (3, 4).

In addition to these pathophysiological processes, mast cells represent a first line of immune defense against pathogens (5), particularly parasites (6), determine immune tolerance (7) and play an important role in wound healing (8) or angiogenesis (9). Proteases released from mast cells are responsible for exogenous toxin inactivation (10).

From direct measurements of mast cell degranulation it is well-known that this process strongly depends on elevation in the concentration of free intracellular calcium ions ([Ca^2+^]_i_) (11). Numerous experiments demonstrate that particularly Ca^2+^ influx from extracellular space is indispensable for mast cell activation [for review see (12)]. Activation of Fcε receptor for IgE (FcεRI) stimulation by antigens is well-described as a trigger to evoke elevation of [Ca^2+^]_i_ in mast cells, but numerous other agonists including adenosine, endothelin 1 (ET-1), Leukotriene C4 (LTC_4_), lysophosphatidylcholine (LysoPC), sphingosine-1-phosphate (S1P) or Substance P have been reported to lead to mast cell activation in a Ca^2+^ dependent manner (12–14). The receptors triggered by these agonists can either potentiate FcεRI-mediated mast cell activation or act by themselves, and stimulate the release of mast cell mediators, using different signaling cascades.

Recently, Mrgprb2 was identified as the target of many small-molecule drugs associated with systemic pseudo-allergic reactions and as the receptor for Compound 48/80, a Ca^2+^ mobilizing mast cell agonist known for years (4). Already in 1974, it was reported that the intravenous administration of Compound 48/80 in dogs triggers an increase of histamine levels in the plasma (15), and some years later, it was shown that Compound 48/80 induces histamine release from rat mast cells (16). Mast cell responses elicited by either FcεRI or Mrgprb2 stimulation differ significantly from each other in many aspects (17, 18), and the molecular constituents of Ca^2+^ entry channels leading to elevation of [Ca^2+^]_i_ following stimulation of Mrgprb2 receptors are unknown. Nevertheless, receptor stimulation leads to a Ca^2+^ influx in rat peritoneal mast cells (19). One type of channel activated by Compound 48/80 is voltage and IP_3_-independent with a 50 pS conductance (20, 21). A more detailed characterization of the inward current through these channels revealed that it has a ratio of Ca^2+^ to Na^+^ permeability (P_Ca_/P_Na_) of 0.55. All seven members of the TRPC subgroup of Transient Receptor Potential (TRP) channel protein family were reported to be expressed in several types of mast cells [for review see (14, 22)]. As TRPC channels represent receptor-operated non-selective cation channels that can be activated by stimulation of various G_q/11_-coupled receptors, it can be speculated whether TRPCs might be involved downstream of Mrgprb2 activation by agonists such as compound 48/80.

Mast cells are classified according to their neutral proteases content: TC mast cells (expressing tryptase and chymotryptic proteinase) and T mast cells (expressing only tryptase) (23) which are also referred as connective tissue-type and mucosal-type mouse mast cells (24). These two distinct mast cells types have different responsivity to different stimuli including Compound 48/80 (25). In the presented work we investigated mast cell activation and Ca^2+^ homeostasis in two classical, well-established models of murine mast cell models: Bone marrow derived mast cells (BMMC) and Peritoneal mast cells (PMC)—representing primary cell models of mucosal and connective tissue type mast cells, respectively. We performed a systematical checkup of different mast cell secretagogues to test their ability to evoke [Ca^2+^]_i_ rise and degranulation in BMMC and PMC. Whereas, antigen stimulation resulted in robust [Ca^2+^]_i_ rise in both BMMC and PMC, we observed much weaker responses with all other agonists in BMMC compared to PMC. PMC showed prominent reactions to Compound 48/80 as well as other Mrgprb2 agonists including Tubocurarine, LL-37 and Substance P. Thus, PMC represent an ideal cell model for comparison of Ca^2+^-dependent mast cell activation elicited by stimulation of FcεRI and by Mrgprb2 receptors. We found an expression of all members of the TRPC protein family in PMC. Experiments using PMC isolated from mice lacking TRPC1, TRPC4, and TRPC6 proteins (*Trpc1/4/6*^−/−^ mice) revealed a functional role of these proteins as constituents of non-selective cation channels activated by Mrgprb2 receptor stimulation, that contribute at least partially to Mrgprb2 receptor-mediated [Ca^2+^]_i_ rise and degranulation in PMC mast cells.

## Methods

### Peritoneal Mast Cell (PMC) Isolation and Culture

Male mice with C57Bl6/N genetic background at the age of 8–14 weeks were used for our experiments. A double knockout mouse line *Trpc1/4*^−/−^ (DKO) and a triple knockout mouse line *Trpc1/4/6*^−/−^ (TKO) was generated by intercrossing mice of the three mouse lines lacking expression of TRPC1 (26), TRPC4 (27), and TRPC6 (28), respectively. Each had been backcrossed to the C57Bl6/N strain (Charles River) for at least 7 generations before they were used to generate the *Trpc1/4*^−/−^ (DKO) and *Trpc1/4/6*^−/−^ (TKO) knockout lines. For comparative Ca^2+^ imaging experiments we used littermatched *Trpc1/4/6*^−/−^ (TKO) and *Trpc1/4*^−/−^; *Trpc*6^+/+^ (DKO) offspring derived from intercrosses of *Trpc1/4*^−/−^; *Trpc*6^+/−^ mice. Animal husbandry and experimental procedures were performed in accordance with local and European Union animal welfare standards.

For the isolation of PMC, peritoneal cells were washed from the peritoneal cavity using peritoneal lavage technique (29). Suspension of peritoneal cells isolated from 2 to 3 mice was pooled together. Cells were centrifuged at ~300 × g and resuspended in RPMI Medium supplemented with 20% Fetal Calf Serum (FCS), 1% PenStrep, 10 ng/ml IL-3, and 30 ng/ml SCF. The cells were further cultured in 5% CO_2_ at 37°C. On the 2nd day of cultivation all non-adherent cells were discarded. The cells were split and transferred into a new flask on day 10. PMC were used for experiments between 14 and 16 days of culturing. Flow cytometry analysis identified 98.5–99.5% cells to be double-positive for FcεRI and c-Kit and could be ranked as mast cells.

### Bone Marrow Derived Mast Cells (BMMC) Culture

Both hind paws were used to dissect femur bones. After cleaning of the bones from connective and muscle tissues, two incisions at distal and proximal sides of each bone were made. Bone marrow was released by two short centrifugation steps (30 s at 5,000 rpm and 120 s at 2,000 rpm). Bone marrow cells were cultured at 1 × 10^6^ cells/ml in IMDM supplemented with 10% heat-inactivated fetal calf serum and 1% penicillin/streptomycin at 37°C, 5% CO_2_. The medium was additionally supplemented with 2 ng/ml IL-3 and 5 ng/ml SCF. The cells were split twice a week and cultured for 6–12 weeks before the experiments (30).

### Expression Analysis

RNA isolation was performed using the RNeasy Mini kit (Qiagen) according to manufacturer's protocol. To avoid false signals originating from genomic DNA, on-column DNA-se digestion was performed and intron spanning RT-PCR primers were used. cDNA synthesis was performed with the SensiFAST cDNA synthesis kit (Bioline) according to manufacturer's instructions. Primers were designed with the Roche® online tool and primer pairs with efficiency between 90 and 110% were used. Quantitative expression analysis was performed using the Universal Probe system (Roche) with the corresponding FastStart Essential DNA Probes Master (Roche) on a LightCycler 96 Instrument (Roche). Expression levels of housekeeping genes (Cxxc1, Aip, H3f3a) were also measured. Primer sequences and probe number for TRPC1 were 5′-ctgaaggatgtgcgagaggt-3′ (fw) and 5′-cacgccagcaagaaaagc-3′ (rev), for TRPC2 were 5′-tccttgtcttcctcggagtc-3′ (fw) and 5′-ttcacagatagggcactggac-3′ (rev), for TRPC3 were 5′-ggtgaactgaaagaaatcaagca-3′ (fw) and 5′-cgtcgcttggctcttatctt-3′ (rev), for TRPC4 were 5′-aaacttttggttcagaaaggtgtc-3′ (fw) and 5′-acagttacagcggacctcgt-3′ (rev), for TRPC5 were 5′-ggcgatgcattactctacgc-3′ (fw) and 5′-gctaagcagaagttccacagc-3′ (rev), for TRPC6 were 5′-aggcaaaaggttagcgacaa-3′ (fw) and 5′-ggcataaaagtcatcttgctgaa-3′ (rev), for TRPC7 were 5′-aatggcgatgtgaacttgc-3′ (fw) and 5′-gtttgattcggctcagacttg-3′ (rev), for Cxcc1 5′-TAGTGCCGACCGCTGACT-3′ (fw) and 5′-GGCCTCTCCCCTAACTGAAT-3′ (rev), for Aip 5′-ACCAGTCATCCACCAAGAGG-3′ (fw) and 5′-AGGCGATGGCGTCATAGTA-3′, for H3f3a 5′-GCCATCTTTCAATTGTGTTCG-3′ (fw) and 5′-AGCCATGGTAAGGACACCTC-3′ (rev).

### Calcium Imaging

For the measurements of the intracellular free Ca^2+^ concentration ([Ca^2+^]_i_) cells were pre-loaded with a ratiometric calcium sensitive dye, Fura-2. For this purpose the cells were incubated 30 min at room temperature in Physiological Salt Solution (PSS) supplemented with 2.5 μM Fura-2 acetoxymethyl ester. PSS composition was (in mM): NaCl 135; KCl 6; MgCl_2_ 1.2; CaCl_2_ 2; HEPES 10; glucose 12. The cells were mounted on the stage of an inverted Fluorescence microscope Axio Observer-A1 (Zeiss Jena, Germany) equipped with 40x Fluar oil Objective (Zeiss, Germany) and imaged using a CCD camera Axiocam MRm5 (Carl Zeiss GmbH, Germany). Cytoplasmic Fura-2 was excited using a light source, Lambda DG-4 Plus (Sutter Instrument, USA). Fluorescence signal was measured at 510 nm during alternate excitation at 340 and 380 nm. The Light Source and the camera were controlled by the Axiovision 4.8.2 software (Zeiss, Germany) through synchronization interface SVB-1 (Zeiss, Germany). After correction for the background fluorescence signals, the fluorescence ratio (F340/F380) was analyzed using Origin (8.5) software (Northampton, USA). For antigen stimulation experiments the PMC were pretreated over night with 300 ng/ml anti-DNP IgE. Experiments were performed at 22–25°C. Ca^2+^ imaging experiments in **Figures 4C–H** and **Figures 4I–N** were performed at different Ca^2+^ imaging setups with different light sources and optical properties of the setups. Therefore, the amplitudes of F340/F380 ratios obtained in *Trpc1/4*^−/−^ DKO PMC vary accordingly and cannot be directly compared between the two sets of experiments.

### Electrophysiological Experiments

Transmembrane currents were measured using EPC- 10 (HEKA Elektronik, Lambrecht, Germany) “patch-clamp” amplifier in the whole-cell configuration. The ramp protocol consisted of a 400-ms ramp from −100 to +100 mV (holding potential of 0 mV) applied at 0.5 Hz. Recordings were started immediately after achievement of whole-cell configuration. The standard extracellular solution for patch-clamp contained in mM: NaCl 135; KCl 6; CaCl_2_ 2; MgCl_2_ 1.2; glucose 12; HEPES 10; pH 7.4, with NaOH. Na^+^ and Ca^2+^-zero extracellular solution contained in mM: NMDG 135; KCl 6; MgCl_2_ 1.2; glucose 10; HEPES 10; pH 7.4 (with HCl). The pipette solution for whole-cell measurements contained in mM: CsCl 80; MgATP 1; creatine 5; glucose 5; HEPES 10; BAPTA [1,2-bis(2-aminophenoxy) ethane-N,N,N,N-tetraacetic acid] 10; CaCl_2_ 4.6; pH 7.4 (with CsOH).

### Beta-Hexosaminidase Release PMC Degranulation Assay

PMC were centrifuged at 300 × g for 5 min and re-suspended in Tyrode solution containing in mM: NaCl 130; KCl 5; CaCl_2_ 1.4; MgCl_2_ 1; glucose 5,6; HEPES 10, 0,1% Bovine Serum Albumine (Fraction V); pH 7.4 (with NaOH). The cells were seeded in a V-bottom 96-well plate (2 × 10^5^ cells/well). All experimental conditions were performed in duplicates. Degranulation was induced by incubation of PMC in the presence of the agonists during 45 min at 37°C and 5% CO_2_. Cells were centrifuged at 300 g for 5 min at 4°C. The supernatants were separated and the cell pellets were lysed in Tyrode solution supplemented with 1% Triton-X 100 during 5 min at room temperature. The amount of released β-hexosaminidase enzyme was quantified by spectrophotometric analysis of 4-Nitrophenyl N-acetyl-β-D-glucosaminide (pNAG) hydrolysis, as previously described (29). In short, the cell lysates and supernatants were incubated separately with 2 mM pNAG for 1 h at 37°C and the reaction was stopped by adding 200 mM glycine (pH 10.7 with NaOH). Hydrolysis rate of pNAG was quantified by colorimetric measurements at 405 nm using the NanoQuant Infinite M200pro (Tecan, Switzerland) spectrophotometer. For the background correction the 630 nm absorbance was subtracted from the 405 nm absorbance values. The percentage β- hexosaminidase release was calculated as the absorbance ratio of the supernatant to the sum of supernatant and lysate. If not stated otherwise, all chemicals used in this work were purchased from Sigma.

### Statistics

For statistical analysis, Origin 8.5 (Northampton, USA) and Microsoft Excel 2010 software were used. For the determination of significant differences of mean values obtained from two groups, a two-sample Student's *t*-test was used (*p* < 0.05 for significance). *n* indicates the number of individual experiments unless otherwise stated.

## Results

### Comparison of Ca^2+^-Dependent MC Activators

**Figure 1:**
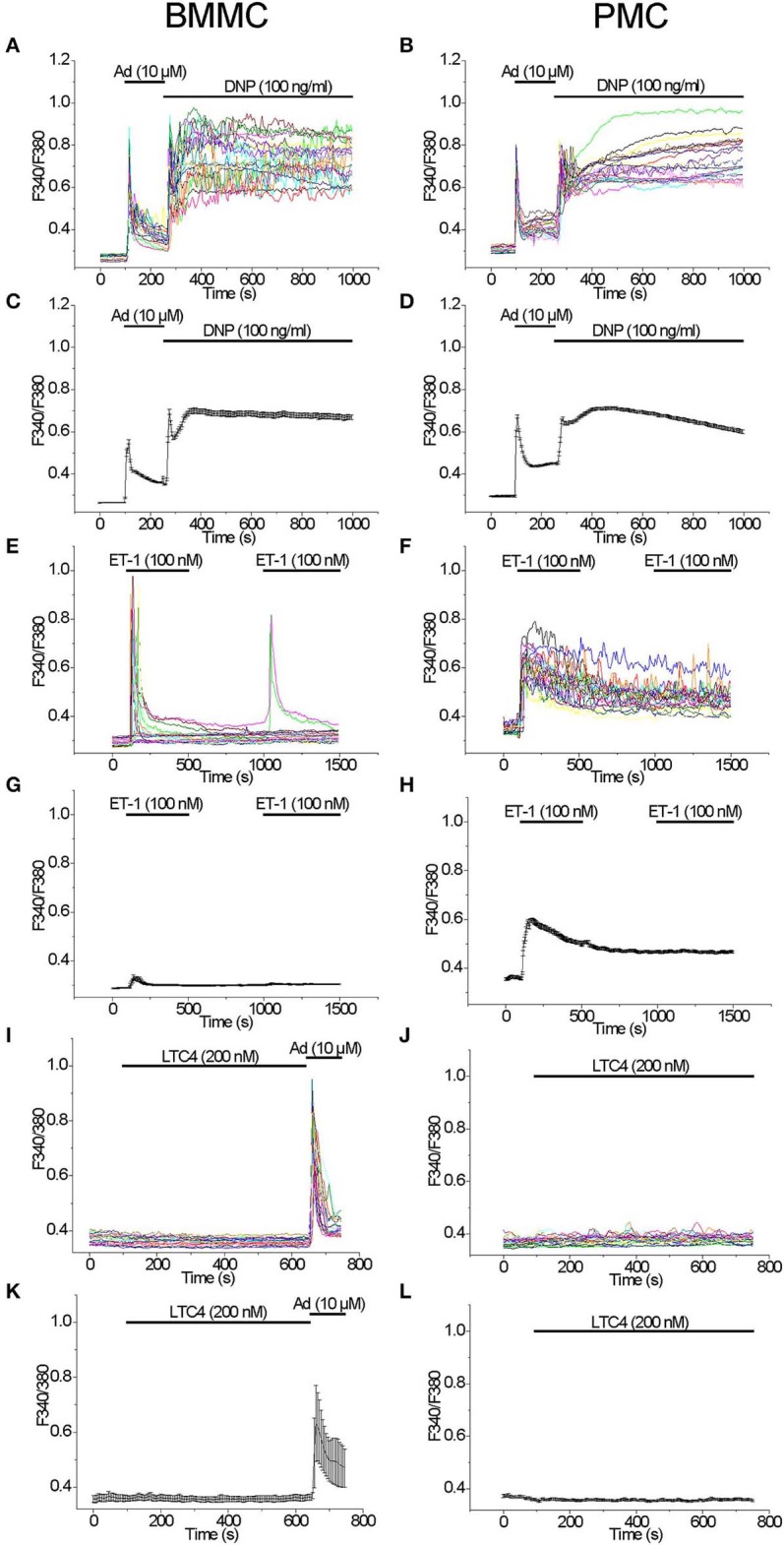
Comparison of [Ca^2+^]_i_ rise induced by different agonists in BMMC and PMC. Measurements of [Ca^2+^]_i_ changes performed with Fura-2 and presented as F340/F380 fluorescence ratio in BMMC **(A,C,E,G,I,K)** and PMC **(B,D,F,H,J,L)** isolated from WT mice. Representative traces (*n* = 20 each panel) of [Ca^2+^]_i_ changes (error bars indicate S.E.M.) induced by application of: 10 μM Adenosine (Ad) and subsequently DNP (100 ng/ml) **(A–D)**, 100 nM Endothelin-1 (ET-1) **(E–H)**, 200 nM LTC4 **(I–L)**. The measurements were performed in 3–5 independent cell preparations. At the end of recordings, control reactions were elicited by application of 10 μM adenosine (Ad) **(I,K)**.

We have compared the ability of well-known secretagogues to elevate [Ca^2+^]_i_ in BMMC and PMC. Application of Adenosine (10 μM) with the subsequent application of the antigen DNP (100 ng/ml) as previously described (30) led to a typical biphasic reaction with comparable amplitudes evoked by either agonist in both BMMC and PMC (Figures 1A–D). Acute application of Endothelin 1 (100 nM) evoked a transient response of high amplitude only in some BMMC (Figure 1E). The probability of response to the second application of Endothelin-1 (100 nM) in BMMC was much lower in comparison to the first one (Figure 1E). In contrast to BMMC, acute application of Endothelin-1 evoked a massive synchronized response of high amplitude in all tested PMC (Figure 1F). The removal of the agonist as well as the recurrent application of the same agonist concentration showed no visible effect (Figure 1F). In average, Endothelin-1 evoked a much more pronounced rise in [Ca^2+^]_i_ in PMC in comparison to BMMC (Figures 1G,H). It is published, that activation of cysteinyl leukotriene type I (cysLT1) receptors with Leukotriene C4 (LTC_4_, 160 nM) in RBL2H3 cells evokes a series of oscillations in [Ca^2+^]_i_ involving calcium release activated Ca^2+^ influx (31). We tested LTC_4_ (200 nM) in both BMMC and PMC but did not observe any rise in [Ca^2+^]_i_ (Figures 1I–L).

### Testing of the Ca^2+^ Mobilizing Ability of Mrgprb2 Receptor Agonists in BMMC and PMC

**Figure 2:**
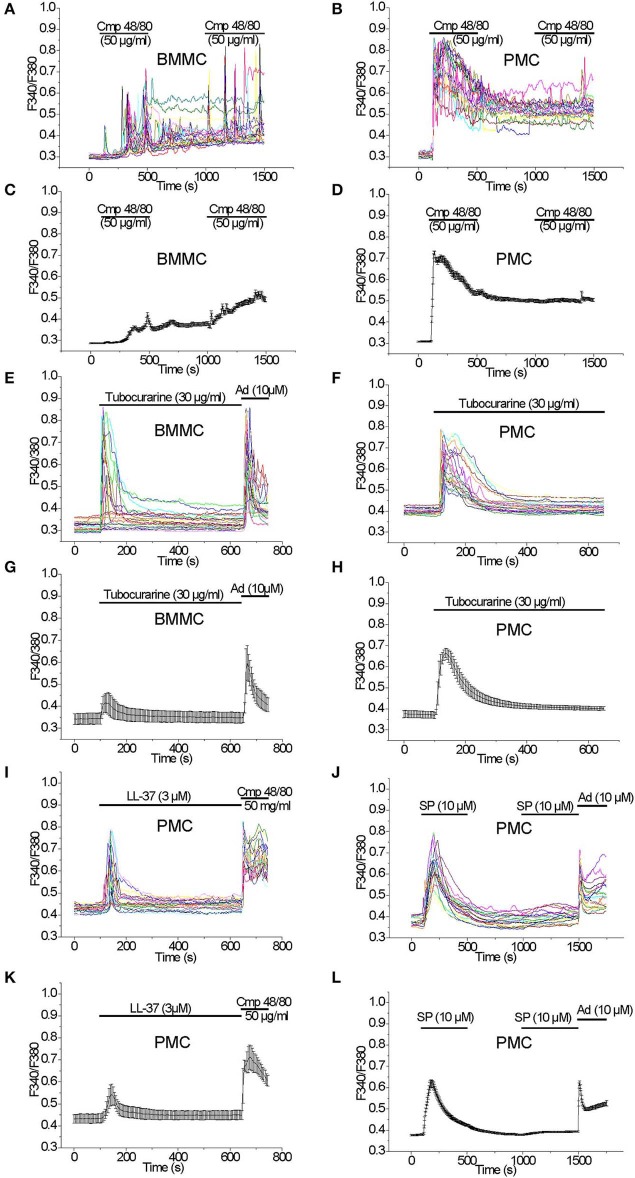
[Ca^2+^]_i_ rise induced by Mrgprb2 agonists in BMMC and PMC. Measurements of [Ca^2+^]_i_ changes performed with Fura-2 and presented as F340/F380 fluorescence ratio in BMMC **(A,C,E,G)** and PMC **(B,D,F,H–L)** isolated from WT mice. Representative traces (*n* = 20 each panel) of [Ca^2+^]_i_ changes (error bars indicate S.E.M.) induced by application of: 50 μg/ml Compound 48/80 **(A–D)**, 30 μg/ml Tubocurarine **(E–H)**, 3 μM LL-37**(I,K)**, 10 μM Substance P **(J,L)**. The measurements were performed in 3–5 independent cell preparations. At the end of recordings, control reactions were elicited by application of 10 μM adenosine “Ad” **(E,G,J,L)** or by application of 50 μM Compound 48/80 “Cmp” **(I,K)**.

Acute application of the Mrgprb2 receptor agonist Compound 48/80 (50 μg/ml) evoked a delayed oscillatory non-synchronous [Ca^2+^]_i_ elevation in BMMC which did not return to the baseline after the agonist removal. A second application of the agonist elicited an additional [Ca^2+^]_i_ elevation (Figures 2A,C). In PMC, acute application of Compound 48/80 (50 μg/ml) evoked an immediate, synchronous and prominent [Ca^2+^]_i_ elevation which also did not get back to baseline levels after the agonist removal. In contrast to BMMC, a second application of the agonist did not elicit an additional [Ca^2+^]_i_ elevation (Figures 2B,D). Based on this robust [Ca^2+^]_i_ rise evoked by Compound 48/80, we tested other Mrgprb2 receptor agonists. Acute application of Tubocurarine (30 μg/ml) evoked an immediate and prominent [Ca^2+^]_i_ elevation in BMMC which was not observed in all tested cells (Figures 2E,G). In PMC, acute application of Tubocurarine (30 μg/ml) evoked an immediate synchronous prominent [Ca^2+^]_i_ elevation which was observed in all tested cells (Figures 2F,H).

Acute application of LL-37 (3 μM), which is an antimicrobial peptide known to activate Mrgprb2 receptors (32), evoked an immediate synchronous [Ca^2+^]_i_ elevation in PMC which was observed in all tested cells (Figures 2I,K). In contrast, application of LL-37 (3 μM) evoked no significant [Ca^2+^]_i_ elevation in BMMC (Figures S1A,B).

Acute application of Substance P (10 μM), which can activate MRGPRX2 receptors (33) in addition to NK receptors (34), evoked an immediate synchronous prominent [Ca^2+^]_i_ elevation in PMC, which was observed in all tested cells. A second application of the agonist did not elicit a significant [Ca^2+^]_i_ elevation (Figures 2J,L). In BMMC, an acute application of Substance P (10 μM) did not evoke a significant elevation of the [Ca^2+^]_i_ (data not shown).

Acute application of the dipeptide L-Carnosine (β-Alanyl-l-histidine) can activate Mrgprd receptors (35) and β-Alanin was reported to activate primary sensory neurons (36). We tested the effect of L-Carnosine in both mast cell types, and found that application of L-Carnosine (1 μM followed by 10 μM) evoked no significant [Ca^2+^]_i_ elevation in BMMC (Figures S1C,E). Also in PMC, no significant [Ca^2+^]_i_ elevation was observed following application of either 1 or 10 μM of L-Carnosine (Figures S1D,F).

### Analysis of Degranulation Evoked by Mrgprb2 Receptor Agonists in Comparison With Other Mast Cells Secretagogues in BMMC and PMC

**Figure 3:**
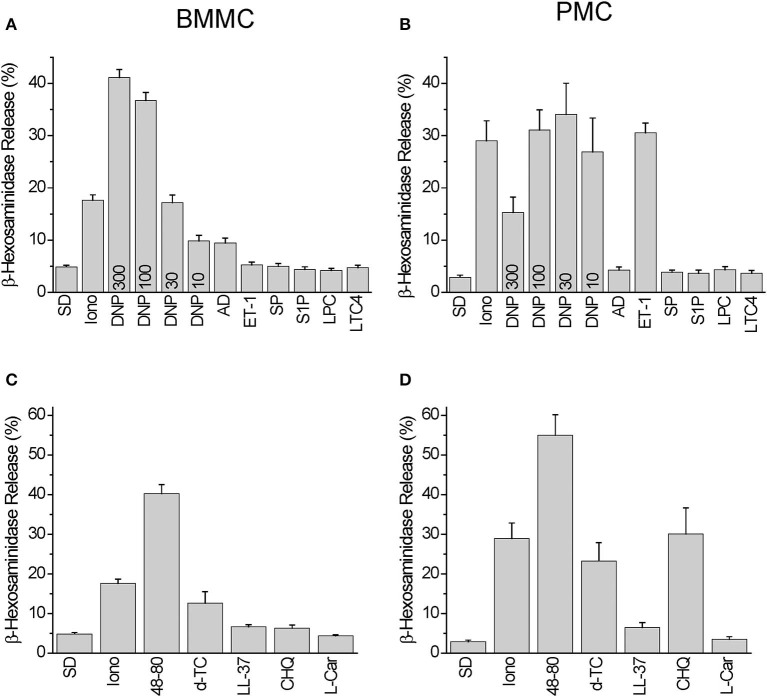
Degranulation induced by Mrgprb2 agonists in BMMC and PMC in comparison with other established mast cell activators. Degranulation of BMMC **(A,C)** or PMC **(B,D)** isolated from wild type C57Bl6/N mice was measured using β-hexosaminidase release assay and presented as % of degranulation. Five **(A,C)** and six **(B,D)** independent preparations were measured. The PMC were stimulated with: vehicle solution (SD- spontaneous degranulation), 10 μM of ionomycin (Iono), 10 ng/ml DNP (DNP10), 30 ng/ml DNP (DNP30), 100 ng/ml DNP (DNP100), 300 ng/ml DNP (DNP300), 10 μM Adenosin (AD), 100 nM Endothelin−1 (ET-1), 10 μM Substance P (SP), 10 μM Sphingosine-1-phosphate (S1P), 10 μM Lysophosphatidylcholine (LPC), 200 nM Leukotriene C4 (LTC4), 50 μg/ml Compound 48/80 (48-80), 30 μg/ml Tubocurarine (d-TC), 3 μM LL-37 (LL-37), 500 μM Chloroquine (CHQ), 300 μM L-Carnosine (L-Car).

Among the Mrgprb2 receptor agonists used in the Ca^2+^ imaging experiments we have observed degranulation reactions in BMMC, which were significantly higher compared to spontaneous degranulation after stimulation with 10–300 ng/ml DNP, 10 μM Adenosine, 50 μg/ml Compound 48/80, and 30 μg/ml Tubocurarine. Degranulation reaction was negligible in response to 100 nM Endothelin-1, 10 μM Substance P, 10 μM Sphingosine-1-phosphate, 10 μM Lysophosphatidylcholine, 200 nM Leukotriene C4, 3 μM LL-37, 500 μM Chloroquine, and 300 μM L-Carnosine, respectively (Figures 3A,C). Stimulation with Ionomycin (10 μM) served as a control.

In PMC, we found degranulation reactions significantly higher than those occurring spontaneously after stimulation with 10–300 ng/ml DNP, 100 nM Endothelin-1, 50 μg/ml Compound 48/80, 30 μg/ml Tubocurarine, 3 μM LL-37, and 500 μM Chloroquine. Degranulation reactions were negligible after application of 10 μM Adenosine, 10 μM Substance P, 10 μM Sphingosine-1-phosphate, 10 μM Lysophosphatidylcholine, 200 nM Leukotriene C4, and 300 μM L-Carnosine, respectively (Figures 3B,D).

Based on these results we can conclude that BMMC exhibit an increased degranulation response compared to PMC following stimulation with 10 μM Adenosine (*p* = 0.007), and PMC exhibit a larger response to 100 nM Endothelin-1 (*p* = 8e-07) and 500 μM Chloroquine (*p* = 0.001). The Mrgprb2 receptor agonist Compound 48/80 evoked similar extents of degranulation in both PMC and BMMC.

### Analysis of TRPC Expression in PMC

**Figure 4:**
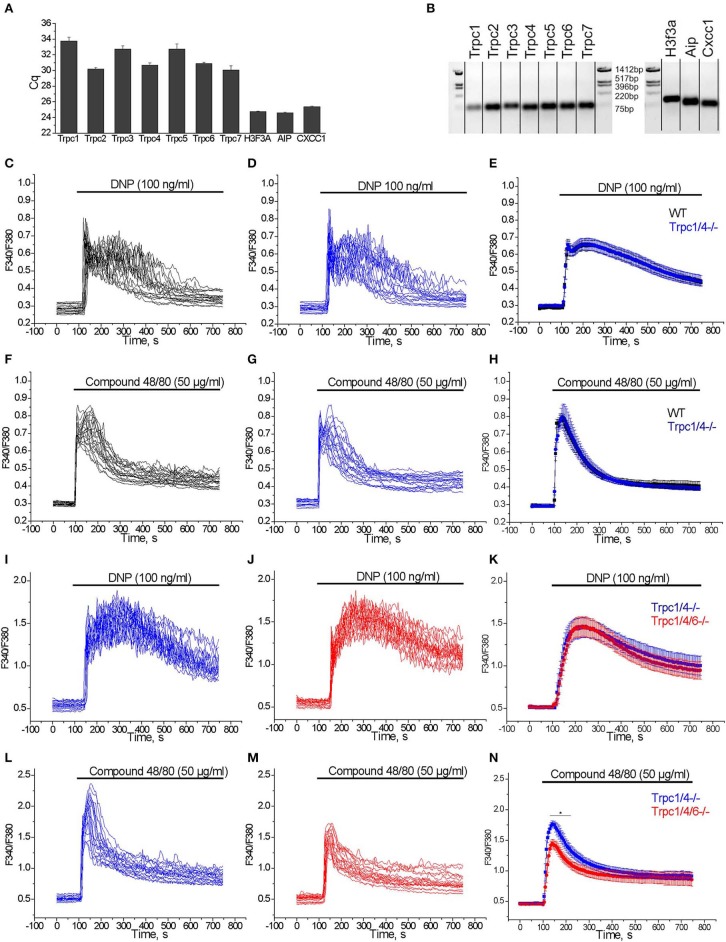
**(A)** RT-qPCR expression analysis of Trpc transcripts in PMC. Cq values (mean ± S.E.M.) were obtained from duplicates of 3 independent wild type PMC preparations. **(B)** RT-qPCR end products are visualized by agarose gel (2%) electrophoresis. The amplicons correspond to the calculated sizes: Trpc1- (65 bp), Trpc2- (87 bp), Trpc3- (75 bp), Trpc4- (60 bp), Trpc5- (63 bp), Trpc6- (61 bp), Trpc7- (64 bp), H3f3a - (90 bp), Aip - (86 bp), Cxcc1 - (66 bp). **(C–H)** Measurements of [Ca^2+^]_i_ changes performed with Fura-2 presented as F340/F380 fluorescence ratio in PMC isolated from WT (C57Bl6/N, black) and *Trpc1/4*^−/−^ (DKO) mice (backcrossed for seven generations on a C57Bl6/N background, blue). Representative traces (*n* = 20) of [Ca^2+^]_i_ changes induced by application of 100 ng/ml DNP **(C,D)** or 50 μg/ml Compound 48/80 **(F,G)** as indicated by horizontal bars. Mean values of [Ca^2+^]_i_ changes (error bars indicate S.E.M.) induced by application of 100 ng/ml DNP **(E)** or 50 μg/ml Compound 48/80 **(H)**. For the statistical analysis an average of mean traces obtained from 4 independent preparations was calculated. In each preparation at least 200 cells were imaged and analyzed. **(I–N)** Measurements of [Ca^2+^]_i_ changes performed with Fura-2 and presented as F340/F380 fluorescence ratio in PMC isolated from *Trpc1/4/6*^−/−^ (TKO) mice (red) and *Trpc1/4*^−/−^ (DKO) littermates (genotype *Trpc1/4*^−/−^; *Trpc6*^+/+^, blue) and. Representative traces (*n* = 20) of [Ca^2+^]_i_ changes induced by application of 100 ng/ml DNP **(I,J)** or 50 μg/ml Compound 48/80 **(L,M)** as indicated by horizontal bars. Mean values of [Ca^2+^]_i_ changes (error bars indicate S.E.M.) induced by application of 100 ng/ml DNP **(K)** or by application of 50 μg/ml Compound 48/80 **(N)**. For the statistical analysis an average of mean traces obtained from 5 **(K)** to 6 **(N)** independent preparations was calculated. In each preparation at least 200 cells were imaged and analyzed.

We studied the expression of transcripts encoding TRPC channel proteins by RT-qPCR in PMC. As shown in Figure 4A, all TRPCs transcripts are detected in PMC, with Trpc2, Trpc4, Trpc6, Trpc7 transcripts being more abundant compared to Trpc1, Trpc3, and Trpc5. The relative expression is presented as Cq values which are reverse proportional to the level of expression. Endpoint PCR products were visualized by gel electrophoresis and amplicons of the expected size were obtained (Figure 4B).

### Partial Contribution of TRPC Channels to [Ca^2+^]_i_ Elevation Elicited by Mrgprb2 Receptor Stimulation in PMC

From former experiments using PMC from various Trpc knockout mouse lines we had evidence that Ca^2+^ elevation evoked by several agonists were reduced in mast cells of *Trpc1/4/5/6*^−/−^ quadruple knockout mice (37), as well as in*Trpc1/4/6*^−/−^ triple and *Trpc1/4*^−/−^ double knockout mice. The mast cells for these experiments had been isolated from mouse lines on a mixed C57Bl6/N × 129SvJ genetic background and compared to PMC of mice from the F1 generation of C57Bl6/N × 129SvJ matings as controls (data not shown). However, when we analyzed calcium transients measured in PMC from *Trpc1/4*^−/−^ double knockout (DKO) mice on C57Bl6/N background (mated from *Trpc1*^−/−^ and *Trpc4*^−/−^ single knockout mice after 7 generation backcrossing) we found that [Ca^2+^]_i_ elevation evoked by FcεRI stimulation by acute application of DNP (100 ng/ml) was statistically not significantly different compared to those in PMC isolated from C57Bl6/N wild type controls (Figures 4C–E). Similarly, Ca^2+^ transients observed in PMC in response to acute application of 50 μg/ml of Compound 48/80 were also statistically not significantly different in PMC isolated from *Trpc1/4*^−/−^ DKO mice in comparison to C57Bl6/N wild type PMC (Figures 4F–H).

Since the responses to FcεRI and Mrgprb2 receptor stimulation were identical in PMC from *Trpc1/4*^−/−^ DKO and WT mice, we then compared PMC from *Trpc1/4/6*^−/−^ (TKO) knockout mice and used littermatched *Trpc1/4*^−/−^ DKO mice as controls (see methods for breeding scheme). Calcium transients evoked in PMC in response to acute application of DNP (100 ng/ml) were statistically not significantly different in PMC isolated from *Trpc1/4/6*^−/−^ (TKO) mice or *Trpc1/4*^−/−^ DKO mice (Figures 4I–K). In contrast, peak calcium transients evoked in response to acute application of Compound 48/80 (50 μg/ml) were significantly lower (by 24%) in PMC from *Trpc1/4/6*^−/−^ (TKO) mice in comparison to those in PMC isolated from *Trpc1/4*^−/−^ (DKO) mice (Figures 4L–N). The AUC of the Ca^2+^ transients was reduced by 20 % in average.

### Characterization of Compound 48/80-Induced Currents in PMC

**Figure 5:**
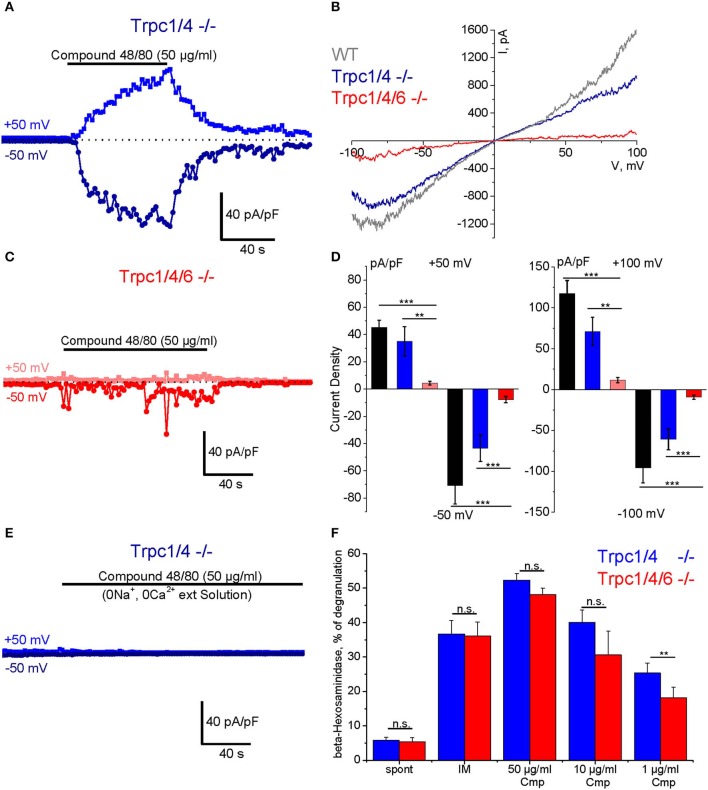
Compound 48/80-induced whole-cell transmembrane currents **(A–E)** and degranulation **(F)** in PMC isolated from TRPC-deficient mice. **(A)** Representative time course of the amplitude of inward (dark blue) and outward (blue) currents measured at −50 and +50 mV, respectively in PMC isolated from *Trpc1/4*^−/−^ (DKO) mice (DKO, genotype *Trpc1/4*^−/−^; *Trpc6*^+/+^). The currents were elicited by acute application of Compound 48/80 (50 μg/ml) as indicated by horizontal bar. **(C)** Representative time course of the amplitude of inward (red) and outward (pink) currents measured at −50 and +50 mV, respectively in PMC isolated from *Trpc1/4/6*^−/−^ (TKO) mice. The currents were elicited by acute application of Compound 48/80 (50 μg/ml) as indicated by horizontal bar. Current amplitude is normalized to the cell capacitance and presented as pA/pF. **(B)** Representative current-voltage relationship curves measured in PMC isolated from wild type (WT, gray), *Trpc1/4*^−/−^ (DKO, genotype *Trpc1/4*^−/−^; *Trpc6*^+/+^, blue) and from *Trpc1/4/6*^−/−^ (TKO) mice (red). The curves correspond to the measurements presented in **(A,C)** at the current's maximal values. **(D)** Statistical bar diagrams of the maximal current density measured at +50/−50 mV (left panel) and at +100/−100 mV (right panel) shows the mean (vertical bars) and S.E.M. (error bars) values calculated for PMC isolated from WT (gray and black bars) Trpc1/4 double deficient (blue and dark blue) and Trpc1/4/6 deficient (pink and red bars) mice. The measurements were performed with PMC originating from three independent preparation and 15–20 individual cells were analyzed. Horizontal bars indicate significance (**p < 0.01 and ***p < 0.001). **(E)** Representative time course of the amplitude of inward (dark blue) and outward (blue) currents measured at −50 and +50 mV, respectively in PMC isolated from *Trpc1/4*^−/−^ (DKO) mice. Compound 48/80 was applied in Ca^2+^- and Na^+^-free external solution in concentration 50 μg/ml as indicated by horizontal bar. **(F)** Degranulation of PMC measured using ß-hexosaminidase release assay and presented as % of degranulation. PMC were isolated from *Trpc1/4*^−/−^ (DKO) mice (blue bars) and *Trpc1/4/6*^−/−^ (TKO) (red bars) mice and stimulated with different concentrations of Compound 48/80 (Cmp). Positive (IM: −10 μM of ionomycine) and negative controls (spont- spontaneous release) were also measured.

To study Compound 48/80-induced transmembrane ionic currents in PMC we applied the standard patch clamp voltage clamp technique in the whole-cell configuration. The cells were perfused with Cs^+^-based pipette solution to block K^+^ channels, and [Ca^2+^]_i_ was strongly buffered at a concentration close to 100 nM. A standard external physiological salt solution was used for these experiments. The cells were kept at a holding potential of 0 mV and periodical 400 ms depolarizing ramp pulses (−100 to +100 mV) were applied at a frequency of 0.5 Hz. In response to external application of Compound 48/80 (50 μg/ml) we observed a slowly developing current, which reached a plateau phase after 1–2 min and showed an almost linear current-voltage relationship and a reversal potential near 0 mV (Figures 5A,B). Agonist removal led to gradual reduction of the current which returned almost to the basal level during 1–3 min (Figure 5A). These currents were observed in PMC isolated from WT (C57Bl6/N, black bars in Figure 5D) as well as from *Trpc1/4*^−/−^ (DKO) mice, and their amplitude was not different in PMC of these two genotypes. However, the additional deletion of TRPC6 proteins in PMC of *Trpc1/4/6*^−/−^ (TKO) mice significantly decreased the amplitude of the inward and outward currents compared to PMC from *Trpc1/4*^−/−^ (DKO) or C57Bl6/N mice as indicated in representative traces (Figure 5C) and in the statistical analysis performed at +50, −50, +100, −100 mV (Figure 5D). The current in PMC from *Trpc1/4*^−/−^DKO mice was also almost entirely abolished if an extracellular solution lacking Na^+^ and Ca^2+^ ions was used (Figure 5E).

### Participation of TRPC6 in Compound 48/80 Induced Degranulation in PMC

A β-hexosaminidase assay was performed in PMC of *Trpc1/4/6*^−/−^ (TKO) knockout mice and litter-matched *Trpc1/4*^−/−^ DKO mice. Whereas, no difference in degranulation was observed when the cells were stimulated with 50 μg/ml and 10 μg/ml Compound 48/80, the measurements revealed a significant reduction (28% in average) of Compound 48/80-induced degranulation in PMC lacking TRPC1/TRPC4/TRPC6 proteins when a concentration of 1 μg/ml of Compound 48/80 was used (Figure 5F).

## Discussion

In this study we comparatively analyzed numerous secretagogues in two primary murine mast cell (MC) models: peritoneal mast cells (PMC), as an example of connective tissue type MC (CTTMCs) (38), and bone marrow-derived and *in vitro* matured mast cells (BMMC) (39) which belong to the mucosal type MCs (MMCs). We provide experimental evidence for at least a partial contribution of TRPC channel proteins to Ca^2+^-dependent mast cell activation in PMC evoked by the Mrgprb2 agonist Compound 48/80.

### Characteristics of CTTMC and MMC Mast Cell Models

With a comparative analysis of two culture-matured and tissue-derived mast cell models we aimed to functionally evaluate the suitability of these primary mast cells for the analysis of Ca^2+^-dependent mast cell activation *in vitro*. The main difference between the connective tissue and mucosal type mast cells is the variety of proteases they express. Thus, mature connective-tissue mast cells express chymases known as mouse mast-cell protease-4 (MMCP-4), -5, and -2, as well as tryptases MMCP-6 and -7, and carboxypeptidase A. Mast cells of the mucosal type preferentially express proteases such as MMCP-1 and -2 as seen in rat mast cells (40, 41). In addition, connective-tissue-type mast cells express little or no NDST-1(*N*-deacetylase/*N*-sulphotransferase-2), but contain large amounts of the transcript encoding NDST-2 (42). It is also known that PMC express a wider range of Toll-like receptors (TLRs), and secrete significantly more cytokines in response to TLR ligand stimulation compared to BMMC or to immortalized mast cell lines (43). The expression of distinct proteases can be well illustrated by the fact that *Mcpt5-Cre* (mast-cell protease-5) transgenic mice enable gene inactivation only in connective tissue mast cells but not in mucosal mast cells (44). In contrast to extensive characterization of connective tissue and mucosal type mast cells with respect to their enzymatic repertoire, there is very little information regarding their responses to the numerous published Ca^2+^ mobilizing mast cell activators. In particular, the activity of these Ca^2+^ mobilizing mast cell activators has not been comparatively characterized in BMMC and PMC as the two primary murine mast cell models (12, 45), which were studied very frequently after gene-deficient mouse lines became increasingly available.

### Features of the Agonist-Evoked Ca^2+^ Rise in BMMC and PMC

An elevation of the [Ca^2+^]_i_ in mast cells, which is a key signal for mast cell activation, can be triggered not only by activation of Fcε receptor for IgEs (FcεRI) with antigens but has also been reported after exposure to numerous agonists such as adenosine, Endothelin-1 (ET-1), Leukotriene C4 (LTC_4_), lysophosphatidylcholine (LysoPC), sphingosine-1-phosphate (S1P) or Substance P and even others (14). In our study we found that, whereas antigen stimulation of FcεRI was similarly effective in triggering [Ca^2+^]_i_ elevations in PMC and BMMC, other agonists including Endothelin-1 (ET-1, 100 nM) evoked much more prominent responses in PMC in comparison to BMMC or did not evoke any [Ca^2+^]_i_ rise in either mast cell model. As expected, the magnitude of [Ca^2+^]_i_ rise correlated with extent of mast cell degranulation. Similar results were obtained in a comparison of Endothelin-1 (ET-1)-induced degranulation in fetal skin-derived cultured mast cells (FSMCs) and BMMC, as ET-1 induced degranulation in FSMC but not in BMMC (46). The cysteinyl leukotriene receptor I (cysLT1) agonist, leukotriene C4, did not produce any [Ca^2+^]_i_ rise or degranulation response neither in BMMC nor in PMC at least in the concentration of 200 nM. This is in contrast to published data that show that LTC_4_ (160 nM) is quite effective in producing [Ca^2+^]_i_ rise in other mast cell models such as RBL2H3 cells (31). Notably, LTC_4_ plays a central role in activation of human nasal polyp–derived mast cells in a paracrine way (47). Obviously, murine BMMC and PMC are not suitable models to study Ca^2+^ dependent mast cell function that are evoked by Cysteinyl leukotriene receptors and triggered by LTC_4_.

### Mrgprb2-Mediated Ca^2+^ Signaling in BMMC and PMC

Compound 48/80 is well-known as a Ca^2+^-dependent mast cell activator for years, but the receptor mediating Ca^2+^ entry and mast cell activation by this agonist and a range of other cationic substances termed basic secretagogues was identified in 2015 (4). We found that the Mrgprb2 agonist Compound 48/80 evoked a significant [Ca^2+^]_i_ rise and degranulation response in PMC and BMMC, but these responses were much weaker in BMMC. Other agonists of Mrgprb2 receptors such as Tubocurarine or LL-37 and Chloroquine, which are reported to activate other Mrgpr receptors (48), demonstrated a similar relation regarding their action in BMMC vs. PMC suggesting that the expression level or coupling of Mrgprb2 receptors or of the signaling molecules downstream differs between these two types of mast cells. Substance P evoked a [Ca^2+^]_i_ rise only in connective tissue type PMC and did not produce any significant degranulation in these cells. The functional target of Substance P in PMC and mast cells in general is not clearly defined. Substance P is able to activate the human ortholog of Mrgprb2, MRGPRX2 (33), but not the mouse Mrgprb2 receptor (49). In addition, Substance P is also capable of stimulating NK receptors (34) which are also expressed in TC mast cells (50).

In general, our data demonstrate a much higher efficiency of Mrgpr agonists in PMC to induce [Ca^2+^]_i_ elevation and degranulation in comparison to BMMC. These results are in agreement with those of a previous report, in which it has been shown that Mrgprb2 is specifically expressed on connective tissue mast cells but not on mucosal mast cells in mice (4).

### Contribution of TRPC Channels to Mrgprb2-Mediated Ca^2+^ Rise in PMC

Our expression analysis revealed that transcripts of all members of the TRPC family of transient receptor potential (TRP) channels could be detected in PMC. Since we had evidence that Ca^2+^ elevation evoked by various agonists leading to Phospholipase C activation were reduced in PMC mast cells of *Trpc1/4/5/6*^−/−^ quadruple knockout mice (37) and also *Trpc1/4/6*^−/−^ triple knockout mice both on a mixed C57Bl6/N × 129SvJ genetic background compared to PMC of corresponding wild type control mice, we subsequently studied *Trpc1/4/6*^−/−^ mice that were obtained after seven generation of backcrosses into the C57Bl6/N background, which were then compared to littermatched *Trpc1/4*^−/−^ controls (see Methods for details of breeding). We found that FcεRI-mediated Ca^2+^ rise was not significantly different in PMC from *Trpc1/4/6*^−/−^ mice compared to PMC from either C57Bl6/N or *Trpc1/4*^−/−^ littermatched controls indicating the importance of the genetic background for studies evaluating Ca^2+^ signaling in murine PMC mast cells. Nevertheless, Compound 48/80-evoked [Ca^2+^]_i_ transients were reduced by ~20% in PMC from *Trpc1/4/6*^−/−^ mice which goes along with the abrogation of Compound 48/80-evoked cation currents and an according reduction of mast cell degranulation. In the past, many studies about the role of TRPC channels in mast cells have been performed using immortalized mast cell lines (14). There are only a few reports about the role of TRPC channels and particularly TRPC1, TRPC4, and TRPC6 in mast cell activation. Altered calcium responses in Lyn-deficient mast cells are at least partially attributed to a Lyn-dependent role in maintaining basal expression of Trpc4 (51). In BMMC from TRPC1 knockout mice, an unexpected increase in antigen-evoked interleukin and TNFα secretion was reported (52). TRPC1 and TRPC6 were found to be expressed in human mast cell lines, but a contribution of Orai and not TRPC channels to FcεRI-mediated calcium signaling was demonstrated recently (53). These data are similar to our findings showing that TRPC6 plays a significant role specifically in Mrgprb2- but not FcεRI-mediated [Ca^2+^]_i_ elevation in murine PMC. In murine mast cells the Orai1 knock out (54, 55) and knock down of Orai1 in human lung mast cells (56) reduced FcεRI-mediated calcium rise and FcεRI-dependent mediator release, respectively, whereas these responses was increased in PMC of Orai2 knock out mice (57).

Our study shows that in PMC from *Trpc1/4/6*^−/−^ mice the FcεRI-mediated Ca^2+^ rise is unchanged and Mrgprb2-mediated Ca^2+^ rise is partially reduced. The signaling events downstream of Mrgprb2 receptor activation that specifically engage these TRPC channels remain unclear, but it needs to be mentioned that there is a growing experimental evidence of important differences between MRGPRX2- and FcεRI-mediated mast cell activation and degranulation (17). Notably, FcεRI-mediated mast cell activation involves a robust inflammatory reaction by synthesis of cytokines and chemokines, and this reaction has been shown to be limited in MRGPRX2-mediated activation (17). Classically, IgE-dependent mast cell activation occurs via FcεRI with the subsequent tyrosine phosphorylation of multiple proteins including phospholipase Cγ1 the activation of which leads to multiple downstream events including an increase in cytosolic calcium levels. There is evidence that Mrgpr activation involves activation of phospholipase C which leads to DAG formation. For example, beta-defensins are proinflammatory pruritogens that activate Mrgprs (58) and act chemotactic for mast cells through a pertussis toxin-sensitive and phospholipase C-dependent pathway (59). Diacylglycerol (DAG) formed by phospholipase C could be a signal activating TRPC3/6 channels (60) also in the plasma membrane of mast cells.

The molecular constituents of the channels engaged following Mrgprb2 receptor activation have not been identified. However, it was reported already long time ago that Compound 48/80 activates a Ca^2+^ influx in rat peritoneal mast cells (17). One type of channel responsible for the Ca^2+^ influx followed Compound 48/80 stimulation is voltage–independent and has a relatively low selectivity for Ca^2+^ over Na^+^ (19–21), which closely resembles the properties of agonist-operated TRPC cation channels. In our study in PMC we observed Compound 48/80–induced currents using patch-clamp recordings. These currents exhibited a nearly linear current-voltage relationship with a reversal potential close to 0 mV resembling typical TRPC-mediated currents described in numerous cell models (61). This current was almost absent when an external solution lacking both Ca^2+^ and Na^+^ ions was used demonstrating the cationic nature of Compound 48/80–induced currents. This current was strongly reduced in PMC from *Trpc1/4/6*^−/−^ mice implying that the Compound 48/80-induced Ca^2+^ elevation might be attributed to the Ca^2+^ influx through TRPC6-containing channels in murine PMC. The contribution of TRPC6 to the compound 48/80-evoked current was elaborated by genetic deletion of TRPC6 expression. The deduced concept of TRPC6 as an indispensable constituent of cation channels activated by compound 48/80 in PMCs could be corroborated in future studies by experimental evidence showing that acute blockage of these currents using TRPC6-specific antagonists such as Pyrazolo [1,5-a] pyrimidines antagonist (62) or BI 749327 (63).

Taken together, our data revealed that PMC are an appropriate mast cell model to study mechanisms of Mrgprb2 receptor-mediated mast cell activation and that TRPC proteins contribute at least partially to the activation of PMC evoked by stimulation of Mrgprb2 receptors. However, the channels conducting most of the Ca^2+^ entry triggered by Mrgprb2 receptor stimulation still remain unknown. PMC express plenty of Ca^2+^-permeable cation channels. We have recently excluded a significant participation of TRPV1, TRPV2, and TRPV4 channels in both FcεRI- and Mrgprb2- mediated activation of PMC (64). At the same time Orai1, Orai2, and Orai3 proteins are abundantly expressed in PMC, and at least Orai2 has a critical role in receptor- and store-operated Ca^2+^entry in murine PMC (57), whereas the contribution of Orai1 and Orai3 proteins in Mrgprb2-evoked mast cell activation in general remains to be identified.

## Data Availability Statement

The datasets generated for this study are available on request to the corresponding author.

## Ethics Statement

The animal study was reviewed and approved by the Regierungspräsidium Karlsruhe, Abteilung 3.

## Author Contributions

VT: experimental design, Ca^2+^ imaging, degranulation assay, patch-clamp, and manuscript writing. AS-L: Ca^2+^ imaging and degranulation assay. JA: Ca^2+^ imaging. CR: qPCR. LB and AD: Trpc1 and Trpc6 mouse models. MF: concept and experimental design, data analysis and interpretation, funding, and manuscript writing.

### Conflict of Interest

The authors declare that the research was conducted in the absence of any commercial or financial relationships that could be construed as a potential conflict of interest.

## Supplementary Material

Figure S1 Mrgprb2 agonists without Ca^2+^ mobilizing effects in BMMC or PMC murine mast cells. Measurements of [Ca^2+^]_i_ changes performed with Fura-2 and presented as F340/F380 fluorescence ratio in BMMC **(A–C,E)** and PMC **(D,F)** isolated from WT mice. Representative traces (*n* = 20 each panel) of [Ca^2+^]_i_ changes (error bars indicate S.E.M.) induced by application of 3 μM of LL-37 **(A,B)**, 1 μM L-Carnosine **(C,E)**, and 10 μM of L-Carnosine **(D,F)**. The measurements were performed in 3–5 independent cell preparations. At the end of recordings, control reactions were elicited by application of 10 μM adenosine “Ad” **(A–C,E)** or by application of 50 μM Compound 48/80 “Cmp” **(D,F)**.
