# Supplementary figures and images for: Analysis of Mrgprb2 Receptor-Evoked Ca2+ Signaling in Bone Marrow Derived (BMMC) and Peritoneal (PMC) Mast Cells of TRPC-Deficient Mice
Source: Front Immunol. 2020 Apr 8;11:564. doi: 10.3389/fimmu.2020.00564 (PMC7156601; doi:10.3389/fimmu.2020.00564)

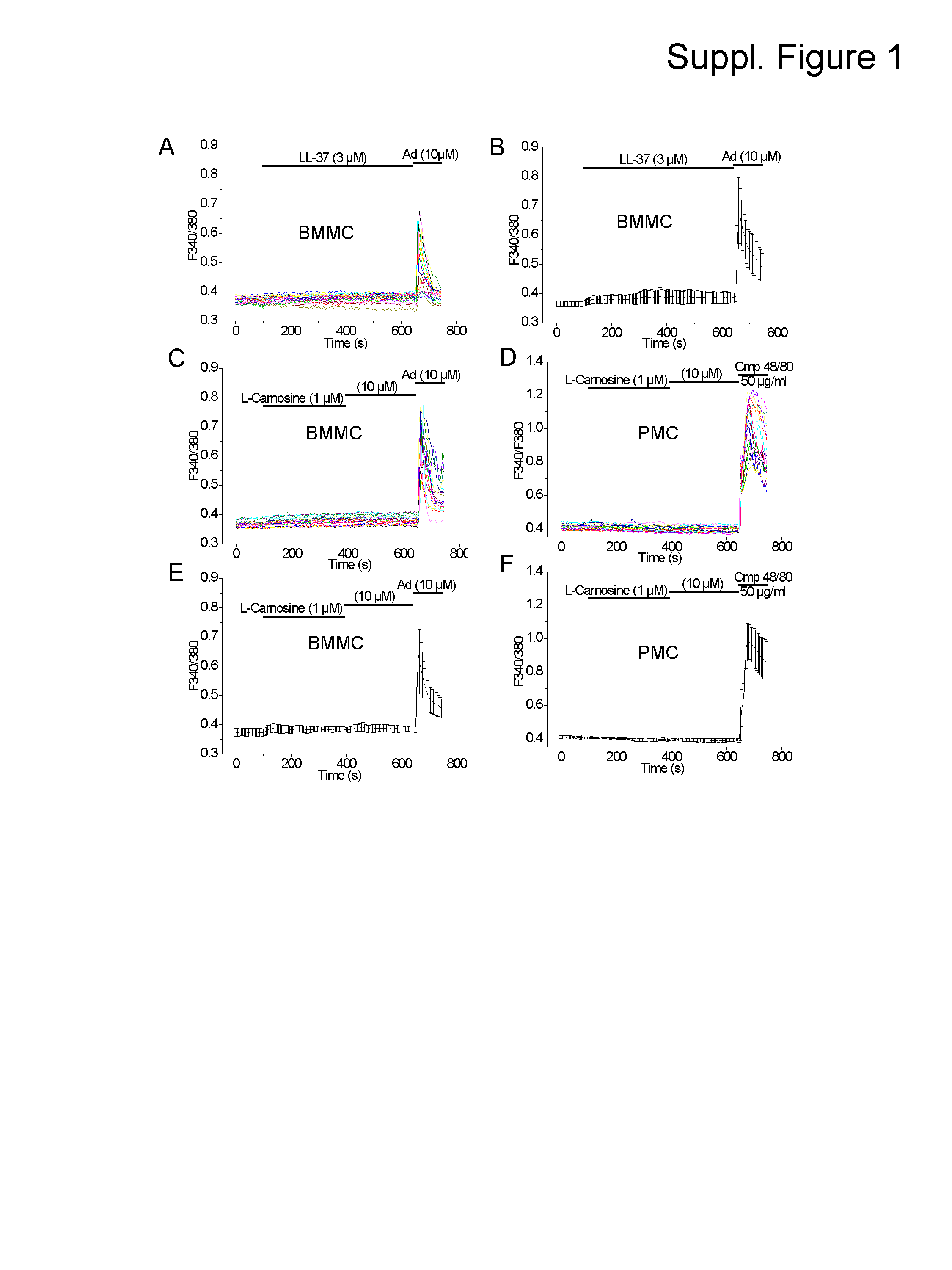

Supplement: Figure S1 — Mrgprb2 agonists without Ca2+ mobilizing effects in BMMC or PMC murine mast cells. Measurements of [Ca2+]i changes performed with Fura-2 and presented as F340/F380 fluorescence ratio in BMMC (A–C,E) and PMC (D,F) isolated from WT mice. Representative traces (n = 20 each panel) of [Ca2+]i changes (error bars indicate S.E.M.) induced by application of 3 μM of LL-37 (A,B), 1 μM L-Carnosine (C,E), and 10 μM of L-Carnosine (D,F). The measurements were performed in 3–5 independent cell preparations. At the end of recordings, control reactions were elicited by application of 10 μM adenosine “Ad” (A–C,E) or by application of 50 μM Compound 48/80 “Cmp” (D,F). [file Image_1.TIF]
